# The Effect of Hypoxic Preconditioning on Induced Schwann Cells under Hypoxic Conditions

**DOI:** 10.1371/journal.pone.0141201

**Published:** 2015-10-28

**Authors:** Ou Chen, Miaomiao Wu, Liangfu Jiang

**Affiliations:** 1 Department of Orthopedic Surgery, Wenzhou Hospital of Integrated Traditional Chinese and Western Medicine, Wenzhou, Zhejiang, China; 2 Department of Orthopedic Surgery, The 2nd Affiliated Hospital & Yuying Children's Hospital of Wenzhou Medical University, Wenzhou, Zhejiang, China; 3 Department of Hand and Plastic Surgery, The 2nd Affiliated Hospital & Yuying Children's Hospital of Wenzhou Medical University, Wenzhou, Zhejiang, China; University of Nebraska Medical Center, UNITED STATES

## Abstract

**Object:**

Our objective was to explore the protective effects of hypoxic preconditioning on induced Schwann cells exposed to an environment with low concentrations of oxygen. It has been observed that hypoxic preconditioning of induced Schwann cells can promote axonal regeneration under low oxygen conditions.

**Method:**

Rat bone marrow mesenchymal stem cells (MSCs) were differentiated into Schwann cells and divided into a normal oxygen control group, a hypoxia-preconditioning group and a hypoxia group. The ultrastructure of each of these groups of cells was observed by electron microscopy. In addition, flow cytometry was used to measure changes in mitochondrial membrane potential. Annexin V-FITC/PI staining was used to detect apoptosis, and Western blots were used to detect the expression of Bcl-2/Bax. Fluorescence microscopic observations of axonal growth in NG-108 cells under hypoxic conditions were also performed.

**Results:**

The hypoxia-preconditioning group maintained mitochondrial cell membrane and crista integrity, and these cells exhibited less edema than the hypoxia group. In addition, the cells in the hypoxia-preconditioning group were found to be in early stages of apoptosis, whereas cells from the hypoxia group were in the later stages of apoptosis. The hypoxia-preconditioning group also had higher levels of Bcl-2/Bax expression and longer NG-108 cell axons than were observed in the hypoxia group.

**Conclusion:**

Hypoxic preconditioning can improve the physiological state of Schwann cells in a severe hypoxia environment and improve the ability to promote neurite outgrowth.

## Introduction

Schwann cells are an important part of the peripheral nerve myelin sheath, and they play an essential role in peripheral nerve regeneration. Schwann cells can release neurotrophic factors to promote the regeneration of peripheral nerves, and they can guide axonal regeneration in the direction of the Bands of Büngner [1,2]. However, the separation and culturing of Schwann cells requires peripheral nerve tissue as a raw material, which can be limited. Moreover, Schwann cells have a long growth cycle and are difficult to amplify. Thus, they are difficult to use for clinical applications.

Bone marrow stem cells are a type of pluripotent cell derived from the mesoderm that can differentiate into osteoblasts, chondrocytes, adipocytes and skeletal myoblasts [3]. Recently, several studies have reported that bone marrow stem cells can differentiate into Schwann cells. In vitro studies have shown that these induced Schwann cells not only have Schwann cell phenotypes, but also that they can promote axonal growth [4,5]. However, all of these studies have been performed at conventional in vitro oxygen concentrations.

The oxygen concentration in vivo is approximately 0.4% [6], which is significantly lower than the 21% oxygen concentration that is conventionally used in vitro. In fact, the majority of seed cells die within the first 24 hours in vivo, an effect that is primarily due to hypoxia-induced seed cell apoptosis [7]. Geng [8] injected bone marrow stem cells into mice with ventricular myocardial infarctions and observed that 99% of the bone marrow stem cells were dead by 4 days later. This result demonstrates that these stem cells are highly susceptible to ischemia and hypoxia. Based on this result, many studies have proposed methods to increase the survival of seed cells under hypoxia [9,10]. Follmar et al. [11] reported that when mesenchymal stem cells (MSCs) transfected with the HO-1 gene were transplanted into mice that had experienced an acute myocardial infarction, by 7 days later, the survival rate of the transplanted cells in the experimental group was 3 times higher than in controls. However, transgenic technology is currently complex, expensive, and not widely used. Greijer and van der Wall [12] demonstrated that the severity of hypoxia influences the level of cell apoptosis vs. survival during hypoxia. For example, 0.5% O_2_ was shown to initiate apoptosis in some cells. To prevent the hypoxia-induced accumulation of genetic mutations, there is a critical balance between pro-apoptotic and anti-apoptotic factors. Hypoxia-inducible factor-1α (HIF-1α) plays an important role in maintaining that balance. Sun et al. [13] simulated hypoxic environments to induce hypoxic preconditioning in bone marrow stem cells, and they found that after hypoxic preconditioning, 1) bone marrow stem cells strongly expressed HIF-1α, 2) apoptosis was decreased, and 3) the loss of mitochondrial membrane potential was minimized. These results indicate that hypoxic preconditioning can have protective effects on the survival of bone marrow MSCs in vivo under ischemic and hypoxic conditions. Upon the exposure of MSCs to hypoxic preconditioning, hypoxia-induced apoptotic pathways are activated quickly, resulting in feedback inhibition of this pathway and thereby reducing apoptosis under subsequent ischemic and hypoxic conditions.

This study examined the protective effects of hypoxic preconditioning and the mechanisms underlying these effects in induced Schwann cells under hypoxic conditions. In addition, this study evaluated the ability of induced Schwann cells exposed to hypoxic preconditioning to promote axonal growth.

## Materials and Methods

Bone marrow stem cells (Cyagen Biosciences Inc., USA), NG-108 cells (ATCC, USA), fetal bovine serum (Gibco, USA), Dulbecco’s Modified Eagle Medium (DMEM)/F12 Culture medium (Gibco), trypsin (Sigma, USA), penicillin (Gibco), streptomycin (Gibco), beta-mercaptoethanol (Amresco, USA), all trans-retinoic acid (Sigma), forskolin (Peprotech, UK), basic fibroblast growth factor (bFGF) (Peprotech), platelet-derived growth factor (PDGF) (Peprotech), recombinant human heregulin-b1 (heregulin-b1) (Peprotech), S-100 antibodies (Abcam, USA), glial fibrillary acidic protein (GFAP) antibodies (Abcam), β-actin antibody (Proteintech Group, USA), RIPA cell lysate (Biotime Company, CHN), BCA protein determination reagent (Biotime Company), SDS PAGE gel preparation kit (Biotime Company), DAB light liquid (Biotime Company), goat anti-rabbit IgG2 (Biotime Company), and Tris-buffered saline plus Tween 20 (TBST) (Biotime Company) were all used for the current study. Annexin V-FITC/PI apoptosis and cell cycle kit(Liankebio company,CHN),Mitochondria Staining Kit(JC-1) (Liankebio company).

### Bone marrow stem cell differentiation into Schwann cells

Anabiotic bone marrow stem cells were suspended in 0.25% trypsin and plated into new dishes at a density of 1 x 10^5^/ml. The cells were maintained at 5% CO_2_ and 21% O_2_ in a 37°C incubator. The media was refreshed every 72 hours, at which time the cells were harvested again using the above steps. Passage 3 bone marrow stem cells were obtained, suspended in 0.25% trypsin and plated into new dishes at a density of 1 x 10^5^/ml. The next day, 1 mM β-mercaptoethanol was added, and the cells were cultured for 24 hours. Then, the medium was removed, and 40 ng/ml all-trans-retinoic acid was added for 72 hours. Finally, the all-trans-retinoic acid was removed, and DMEM/F12 containing 15% fetal bovine serum, 14 μm forskolin, 10 ng/ml bFGF, 5 ng/ml PDGF and 200 ng/ml recombinant human heregulin-b1 was added. The cells were cultured in this medium for 2 weeks, and fresh medium was added every 72 hours.

### Immunofluorescence staining

The differentiated Schwann cells were fixed in 4% paraformaldehyde for 30 minutes. Goat serum was used to block the nonspecific sites. Primary rabbit anti-rat GFAP (1:300) and rabbit anti-rat S-100 antibodies (1:100) were added to the cells and incubated for 12 hours at 4°C. Fluorescein isothiocyanate (FITC)-conjugated goat anti-rabbit IgG (1: 200, Sigma, USA) was added as a secondary antibody, and the specimens were counterstained with DAPI and incubated at room temperature for 1 hour. The positively stained cells were counted in 10 random fields (x 200) under an Olympus fluorescence microscope.

### Experimental groups and cell culture conditions

After the differentiation process was completed, the induced Schwann cells were divided into 3 groups. In group A, the conventional oxygen group, the induced Schwann cells were cultured in 5% CO_2_ and 21% O_2_ at 37°C for 24 hours. In group B, the hypoxia-preconditioning group, the induced Schwann cells were cultured in 5% CO_2_ and 5% O_2_ at 37°C for 24 hours before being transferred to 5% CO_2_ and 0.5% O_2_ at 37°C for 24 hours. In group C, the hypoxia group, the induced Schwann cells were cultured in 5% CO_2_ and 0.5% O_2_ at 37°C for 24 hours.

### Electron microscopy analysis

Schwann cells were collected in groups using 0.25% trypsin and fixed in 2.5% glutaraldehyde for 2 hours. The cells were then rinsed in 0.1 mol/L phosphate-buffered saline (PBS) for 10 minutes, immersed in 1% osmic acid for 2 hours and rinsed in 0.1 mol /L PBS for 10 minutes. The Schwann cells were then serially dehydrated in increasing concentrations of ethanol, filtered and embedded in Epon 812 (Ted Pella, CA, USA). Finally, 70 nm-thick cross-sections were obtained and stained with uranyl acetate and lead citrate for observations of the ultrastructure of the cells.

### Mitochondrial membrane potential analysis

Schwann cells were collected in groups using 0.25% trypsin. Then, 200 μl 10 mg/L JC-1 fluorescent dye was added, followed by light staining for 30 min and centrifugation for 10 minutes at 1000 r/min with a 15 cm radius. The cells were then suspended in 250 μl PBS, and flow cytometry was used to measure mitochondrial membrane potential.

### Apoptosis analysis

The induced Schwann cells were digested with 0.25% trypsin. Then 100 μl PBS was added, and the cells were centrifuged for 10 minutes at 1000 r/min with a 15 cm radius. The supernatant was removed according to the kit instructions, and the cells were rinsed and incubated with 195 μl Annexin V-FITC for 30 minutes at 4°C. Then, the cells were incubated with 5 μl PI and immediately analyzed on a FACSC-LSR.

### Western blot analysis

The induced Schwann cells were collected and washed with PBS. They were then scraped into 50 μl cell lysis buffer containing 100 mM PIPES, 5 mM MgCl_2_, 20% (v/v) glycerol, 0.5% (v/v) Triton X-100, 5 mM EGTA and protease inhibitors (Sigma, UK) on ice for 5 min to extract intracellular proteins. The protein concentrations were subsequently determined, and equal amounts of protein were separated on a 12% gradient acrylamide gel. The proteins were then transferred electrophoretically to polyvinyl difluoride membranes. The membranes were then blocked in 5% non-fat dry milk for 10 minutes in TBST (10 mM Tris, pH 7.5, 100 mM NaCl, 0.1% (v/v) Tween). Then, the membranes were incubated with either monoclonal anti-Bcl-2 (1:500) or monoclonal anti-Bax (1:500) at 4°C overnight. Goat anti-rabbit IgG (1:20000) was used as the secondary antibody and was incubated for 1 hour at 37°C. The membranes were then treated with enhanced chemiluminescence (ECL) for 3 minutes and exposed using OLYMPUS light-sensitive film. A β-tubulin antibody (1:1000; Abcam, UK) was used as an internal protein control. The Quantity One software (Bio-Rad, USA) was used to analyze the protein level in each lane.

### Co-cultures of induced Schwann cells and NG-108 cells

NG-108 cells were collected and plated at a density of 1 x 10^5^/ml per slide flask and allowed to settle for 24 hours. Induced Schwann cells were inoculated into Transwell chambers that were inserted into the membranes (aperture: 0.45 μm, diameter: 24.5 mm) and placed on the slides containing the cultured NG-108 cells for 24 hours in 5% CO_2_ and 0.5% O_2_ at 37°C. The cells were fixed in 4% paraformaldehyde for 30 minutes. Goat serum was used to block the nonspecific sites. The primary mouse anti-rat NEFL antibody (1:500) was added to the cells for 12 hours at 4°C. A FITC-conjugated goat anti-mouse IgG (1:200) antibody was used as a secondary antibody, and the specimens were counterstained with DAPI and incubated at room temperature for 1 hour. The positively stained cells were counted in 10 random fields (x 200) under an Olympus fluorescence microscope.

### Statistical analysis

All data are expressed as the mean ± SD and analyzed using the SPSS 13.0 software. Intergroup differences were tested using one-way analysis of variance (Kruskal–Wallis, non-parametric ANOVA), and pairwise comparisons between each group were analyzed using paired t-tests. The level of significance was set at p < 0.05.

## Results

**Fig 1:**
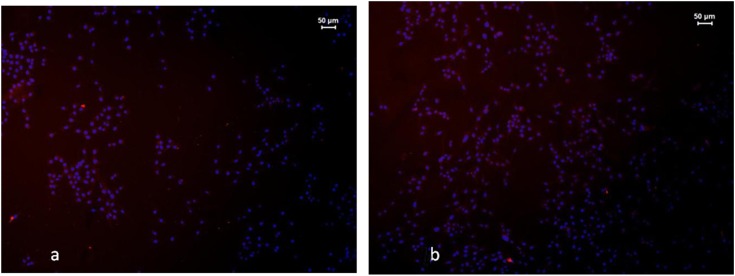
a. Positive expression of S-100 in induced Schwann cells. b. Positive expression of GFAP in induced Schwann cells.

Immunofluorescence staining revealed that 95% of the induced cells were S-100 positive and 92% were GFAP positive, demonstrating that the induced cells were Schwann cells (Fig 1).

**Fig 2:**
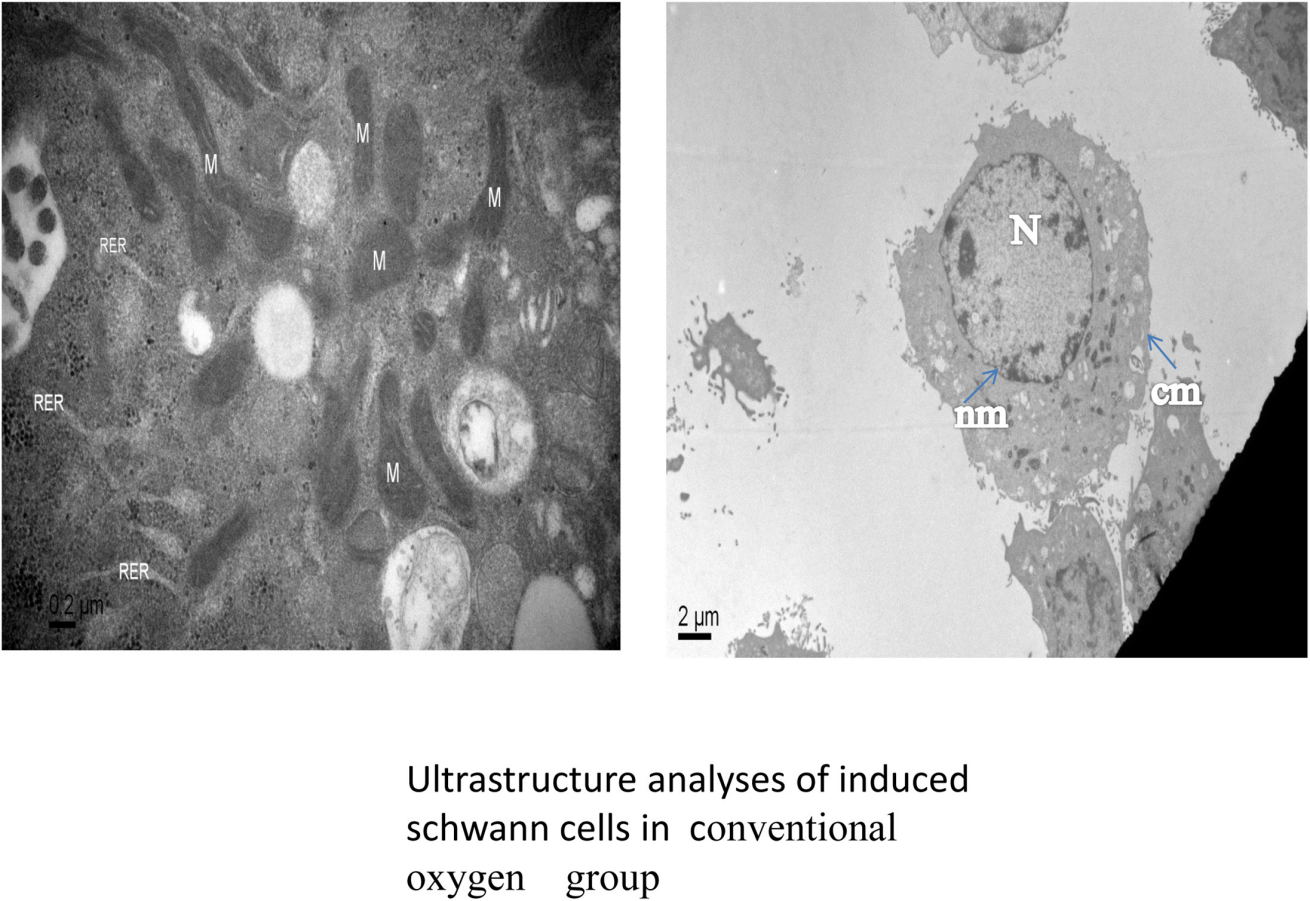
Images from the conventional oxygen group show no cell edema and good cell membrane integrity. Rough endoplasmic reticulum is not dilated, visibly intact mitochondrial membranes, and mitochondria with clear cristae and without swelling and fracturing, the nucleolus is not shrinkage, nuclear membrane integrity. M: mitochondria RER:rough endoplasmic reticulum N:nucleolus, nm: nuclear membrane, cm: cell membrane.

**Fig 3:**
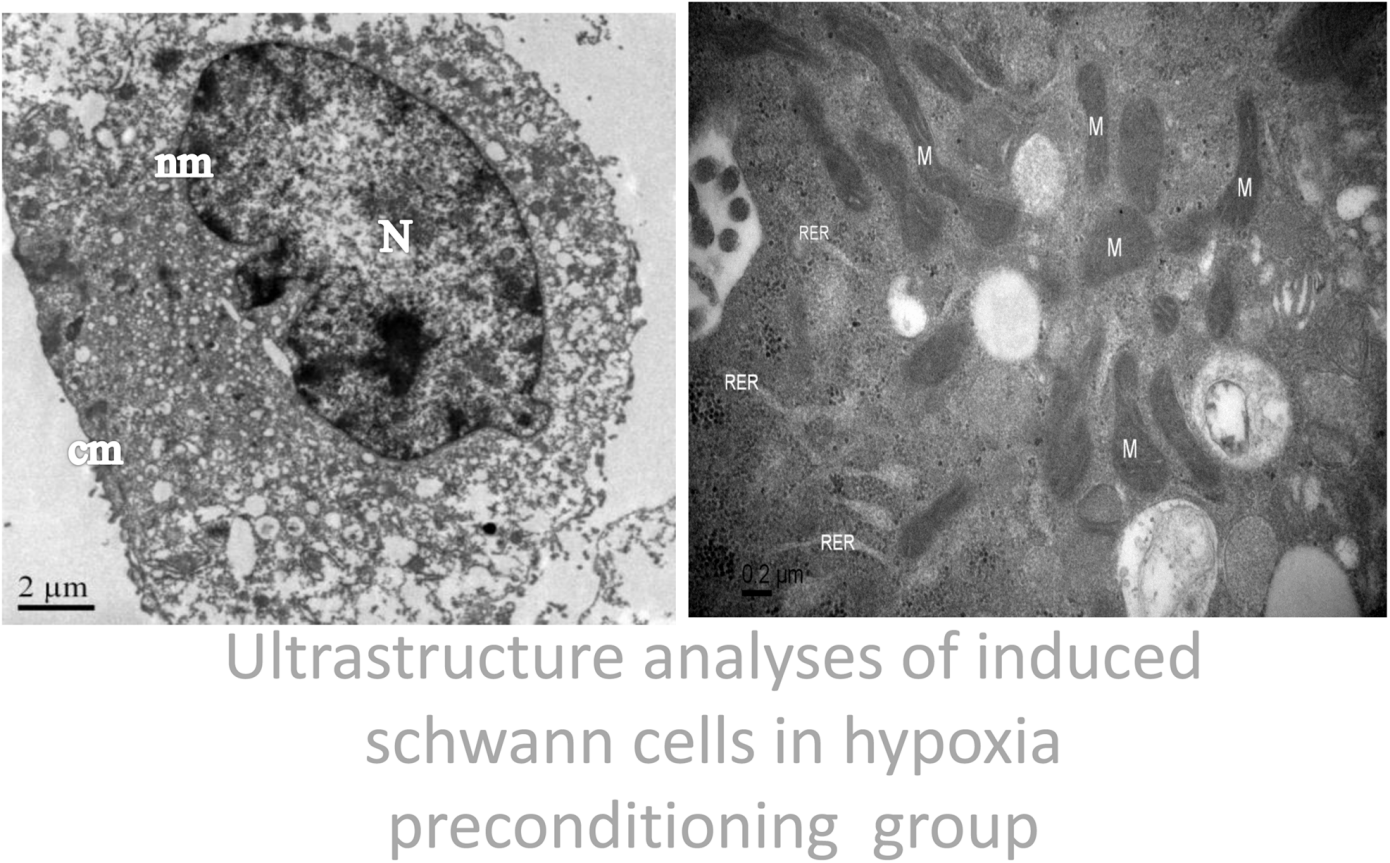
Images from the hypoxia-preconditioning group reveal cell swelling, no obvious cell membrane disintegration, no obvious swelling of mitochondria, mitochondrial cristae with clear and complete structures, Rough endoplasmic reticulum is not dilated, partial nucleolus shrinkage, nuclear membrane integrity. M: mitochondria RER:rough endoplasmic reticulum N:nucleolus, nm: nuclear membrane, cm: cell membrane.

**Fig 4:**
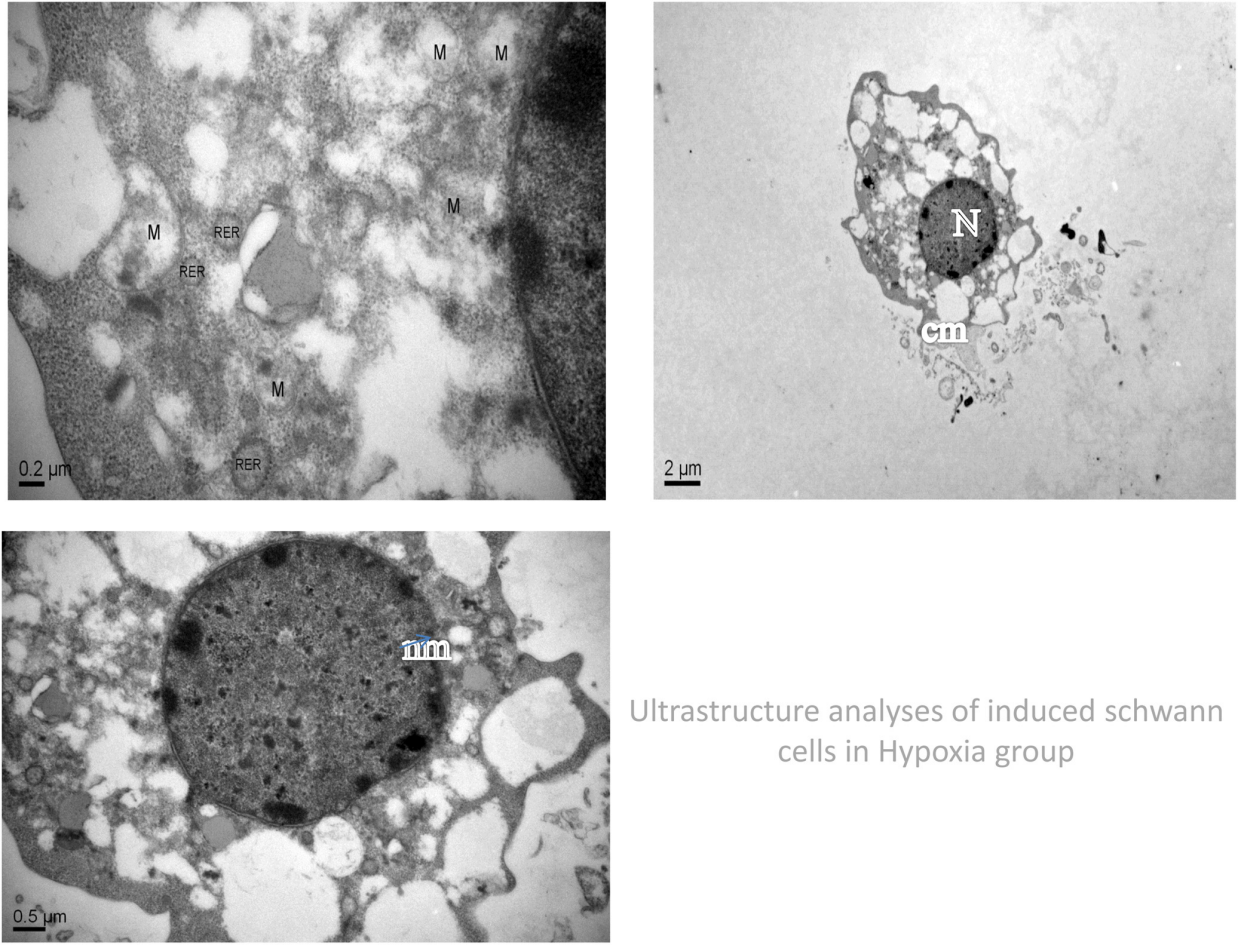
Images from the hypoxia group reveal partial cell membrane disintegration, the whole cell vacuole. organelle fragmentation and disintegration, lysosomal vesicles appear, swelling of the rough endoplasmic reticulum and mitochondria, mitochondria vacuole, mitochondrial cristae shortening and rupture, partial swelling and rupturing of the nuclear membrane, and the nucleolus shrinkage. M: mitochondria RER:rough endoplasmic reticulum N:nucleolus, nm: nuclear membrane, cm: cell membrane.

**Fig 5:**
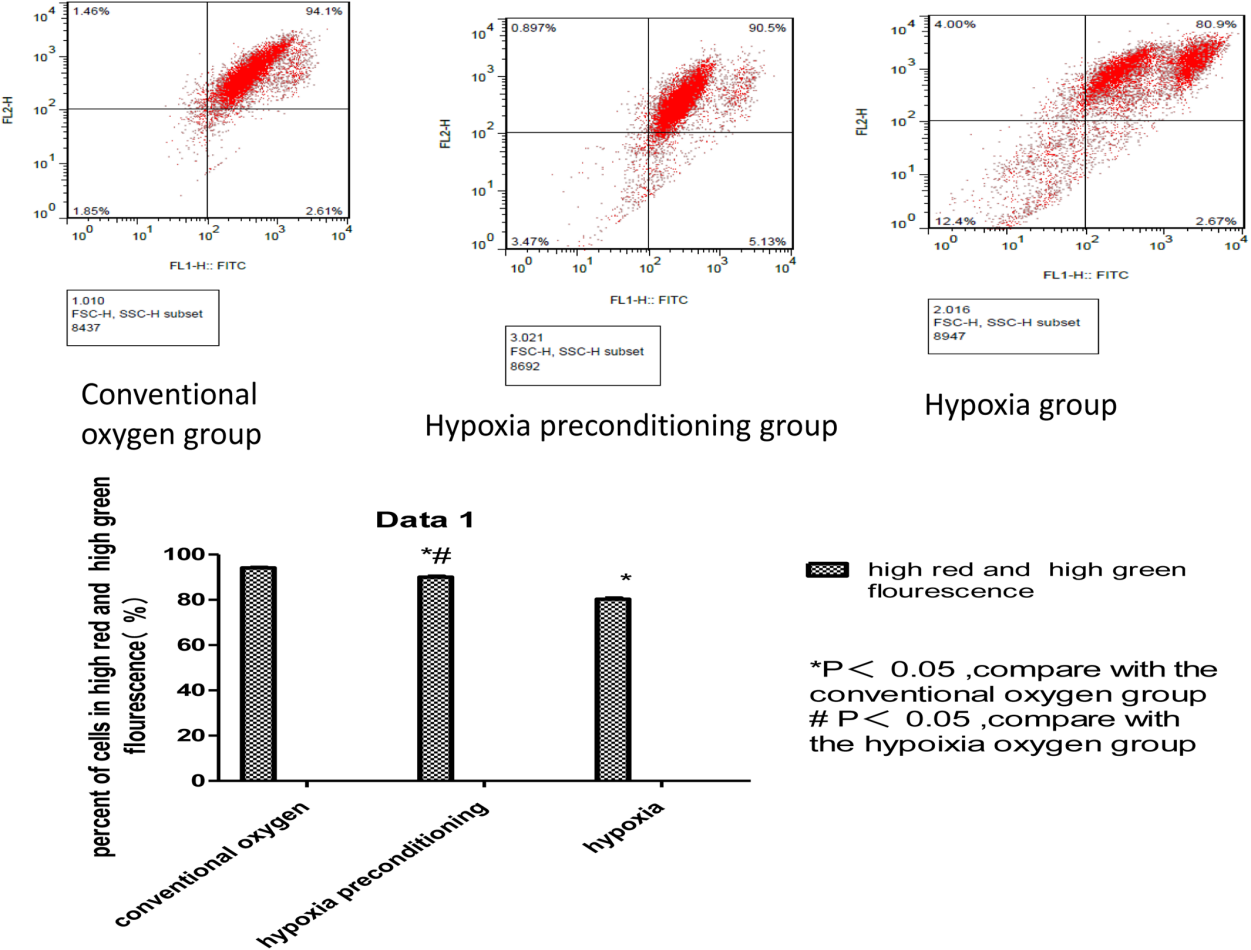
The figure represent the mitochondrial membrane potential of induced Schwann cells in different groups. The points located in the upper-right quadrant represent the number of induced Schwann cells whose mitochondrial membrane potential is not declined. In conventional oxygen group, 94.1±0.2% of the tested induced Schwann cells were in the upper-right quadrant, the hypoxia-preconditioning group, 90.1±0.1% of the tested induced Schwann cells were in the upper-right quadrant, the hypoxia group, 80.6±0.6% of the tested induced Schwann cells were in the upper-right quadrant. The conventional oxygen group had highest ratio of induced Schwann cells whose mitochondrial membrane potential is not declined, and the hypoxia group had lowest ratio of induced Schwann cells whose mitochondrial membrane potential is not declined.

Ultrastructure analyses using an electron microscope revealed that in the conventional oxygen group, the induced Schwann cells exhibited an intact cell membrane, organelles with clear outlines, rough endoplasmic reticulum is not dilated, visibly intact mitochondrial membranes, the nucleolus is not shrinkage, and mitochondria with clear cristae and without swelling and fracturing. In the hypoxia-preconditioning group, the induced Schwann cells exhibited cell swelling, no obvious cell membrane disintegration, no obvious swelling of mitochondria, mitochondrial cristae with clear and complete structures, rough endoplasmic reticulum is not dilated, partial nucleolus shrinkage. In the hypoxia group, the induced Schwann cells exhibited partial cell membrane disintegration, the whole cell vacuole. organelle fragmentation and disintegration, lysosomal vesicles appear, swelling of the rough endoplasmic reticulum and mitochondria, mitochondria vacuole, mitochondrial cristae shortening and rupture, partial swelling and rupturing of the nuclear membrane, and the nucleolus shrinkage. (Figs 2–4). To study the role of mitochondrial dysfunction in Schwann cell apoptosis, we used the lipophilic dye JC-1, which forms red fluorescent aggregates in polarized mitochondria and disperses into green fluorescent monomers as membrane potential decreases, to indicate changes in mitochondrial membrane potential through flow cytometry analysis. On the flow cytometry graphs, the abscissa represents green FITC, and the ordinate represents red PE. In normal cells, the mitochondrial membrane potential is high, indicated by high green and high red color, located in the upper-right quadrant. By contrast, when cells are undergoing apoptosis or are damaged, the mitochondrial membrane potential declines, indicated by low red and high green, causing the number of cells in the upper-right quadrant to decrease and the number of cells in the lower-left quadrant to increase. By comparing the percentage of cells in the upper-right quadrant, we found that the conventional oxygen group had more cells in this quadrant (94.1±0.2%) than the hypoxic preconditioning group (90.1±0.1%) or the hypoxia group (80.6±0.6%) and that these differences were significant (P<0.05). In addition, the percentage of cells in the upper-right quadrant in the hypoxia-preconditioning group (90.1±0.1%) was higher than in the hypoxia group (80.6±0.6%), which was also significant (P<0.05) (Fig 5, Table 1).

**Table 1:** 

Group	The percent of cells in the upper-right quadra(high red and high green fluorescence)
conventional oxygen group	94.1±0.2%
hypoxic preconditioning group	90.1±0.1% * #
hypoxia group	80.6±0.6%*

Value are mean±SD

*P<0.05, compare with the conventional oxygen group

# P<0.05, compare with the hypoixia oxygen group

**Fig 6:**
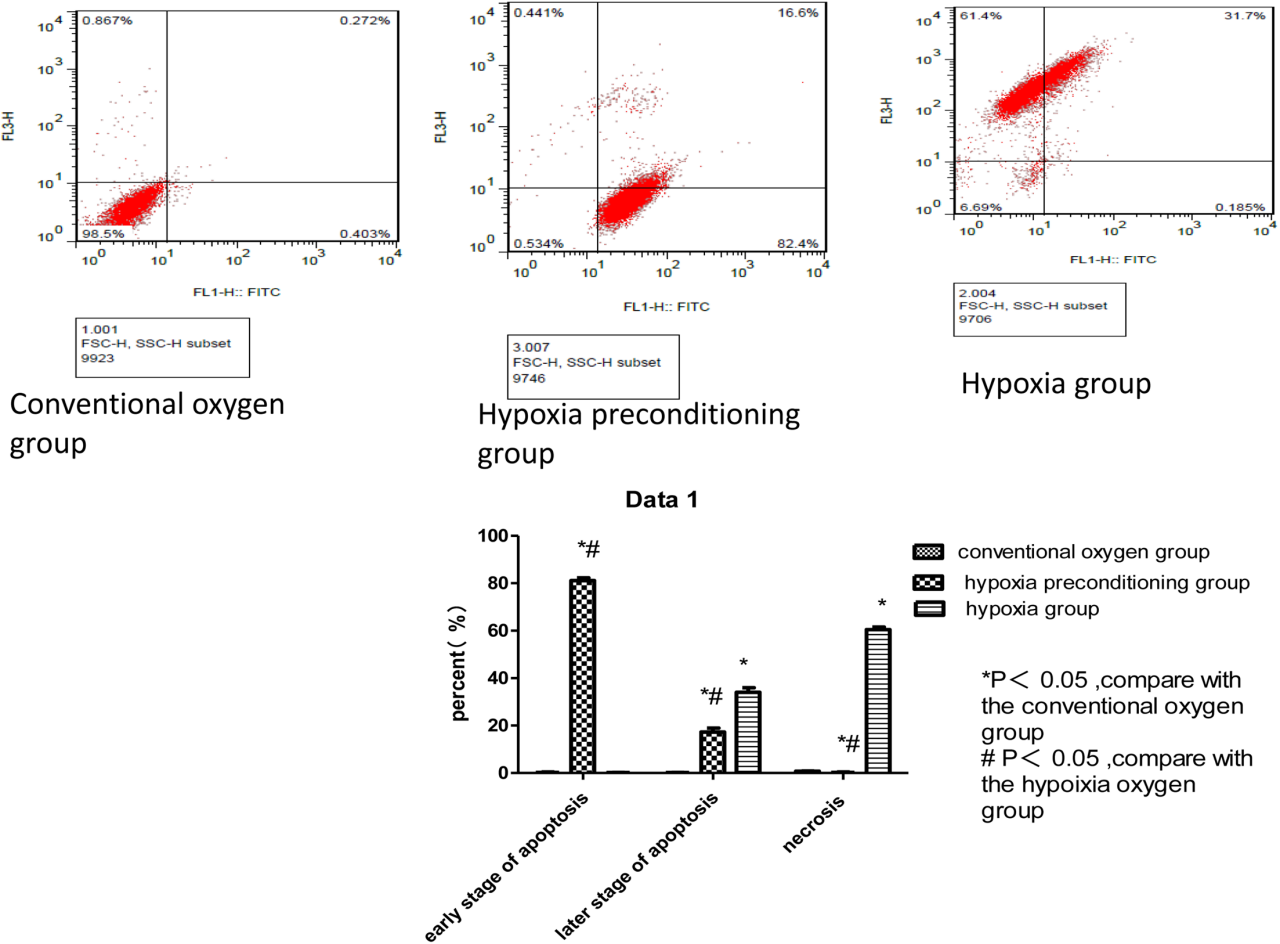
The figure represent the apoptosis rate potential of induced Schwann cells in different groups. There were four quadrants. The high-left quadrant represent the necrosis cells, the low-left quadrant represent the normal cells, the high-right quadrant represent the cells at early stages, and the low-right quadrant represent the cells at late stages. In the conventional oxygen group, most of the induced Schwann cells were not apoptosis or necrosis, the figure showed most of them located in the low-left quadrant.While In the hypoxia-preconditioning group, most cells were in the high-right quadrant,and In the hypoxia group, most cells were in the high-right quadrant. It showed that most cells in hypoxia-preconditioning group were at early stage of apoptosis and most cells in hypoxia group were at late stage of apoptosis.

**Fig 7:**
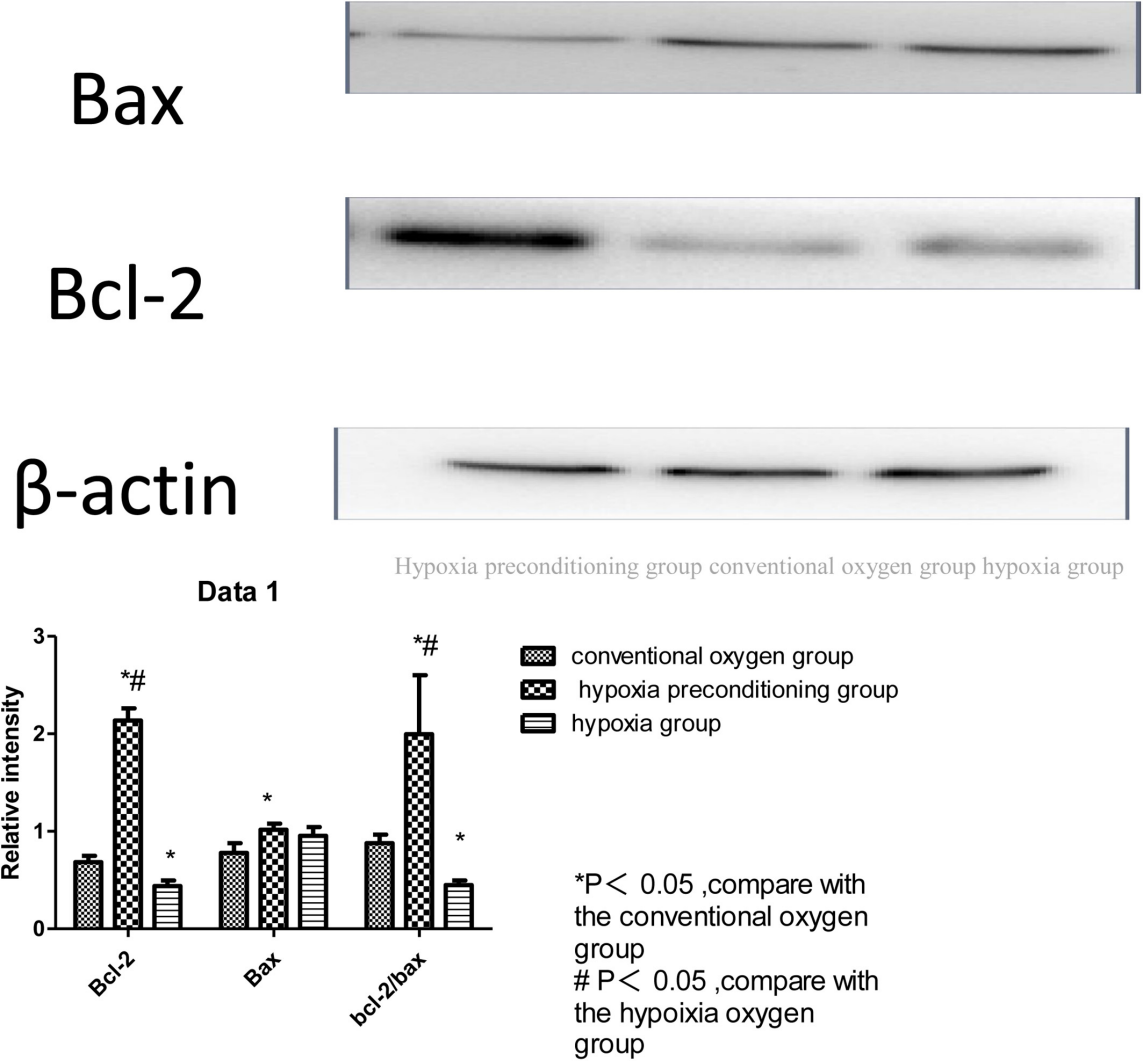
Bax and Bcl-2 expression using Western blot analysis. The figure showed that the hypoxia-preconditioning group had the higher expression of Bax than that in conventional oxygen group,the different was significant,but the different between the hypoxia-preconditioning group and hypoxia group or between the conventional oxygen group and hypoxia group were not significant.While the hypoxia-preconditioning group had the highst expression of Bcl-2, and the hypoxia group had the lowest expression of Bcl-2, the different among the three group were significant. Due to the ratio of Bcl-2/bax, the hypoxia-preconditioning group had the highst expression of Bcl-2/bax, and the hypoxia group had the lowest expression of Bcl-2/bax, the different among the three group were significant.

Using flow cytometry analysis of apoptosis in the hypoxia group, we found that the Schwann cell necrosis rate (60.5 ± 1.81%) was significantly higher than in the hypoxia-preconditioning group (0.27 ±0.07%) or the conventional oxygen group (0.76 ± 0.007%), which was statistically significant (P<0.05). Schwann cells in the later stage of apoptosis in the hypoxia were significantly more common (33.8 ± 3.12%) than in the hypoxia-preconditioning group (17.1 ± 0.70%) or the conventional oxygen group (0.18 ± 0.004%) (P<0.05). In the hypoxia-preconditioning group, the majority of cells were in the early stage of apoptosis (81.6 ± 1.20%), higher than in the conventional oxygen group (0.30 ± 0.12%) or the hypoxia group (0.22 ±0.063%) (P<0.05) (Fig 6A–6E, Table 2). Using Western blot assays, we found that expression of Bcl-2 in the hypoxia-preconditioning group cells was significantly higher (2.14 ± 0.13) than in the conventional oxygen group cells (0.68 + 0.08) and hypoxia group cells (0.46 ± 0.06) (P<0.05). Expression of Bax in hypoxia-preconditioning group cells was also significantly higher (1.02 ± 0.24) than in conventional oxygen group cells (0.78± 0.12) (P<0.05), although not significantly higher than in hypoxia group cells (0.93 ± 0.18) (P>0.05). The Bcl-2/Bax ratios in the conventional oxygen group and the hypoxia-preconditioning group were significant higher than that observed in the hypoxia group, a difference that was significant(P<0.05). The Bcl-2/Bax ratio in the hypoxia-preconditioning group was also higher than that in the conventional oxygen group, but the difference was not significant (Fig 7, Table 3).

**Table 2:** 

Group	necrosis rate	the later stage of apoptosis	early stage of apoptosis
conventional oxygen	0.76 ± 0.007%	0.18 ± 0.004%	0.30 ± 0.12%
hypoxia-preconditioning	0.27 ±0.07% * #	17.1 ± 0.70% * #	81.6 ± 1.20%* #
hypoxia	60.5 ± 1.81%*	33.8 + 3.12% *	0.22 ±0.063%

Value are mean±SD

*P<0.05, compare with the conventional oxygen group

# P<0.05, compare with the hypoixia oxygen group

**Table 3:** 

Group	Bax	Bcl-2	Bax/Bcl-2	the length of NG-108 cell axon (μm)
conventional oxygen	0.78± 0.12	0.68 + 0.08	0.88±0.07	50± 6.39
hypoxia-preconditioning	1.02 ± 0.24*	2.14 ± 0.13* #	2.00±0.49 * #	90 ± 5.28* #
hypoxia	0.93 ± 0.18	0.46 ± 0.06*	0.45±0.03*	128 ± 8.35 *

Value are mean±SD

*P<0.05, compare with the conventional oxygen group

# P<0.05, compare with the hypoixia oxygen group

**Fig 8:**
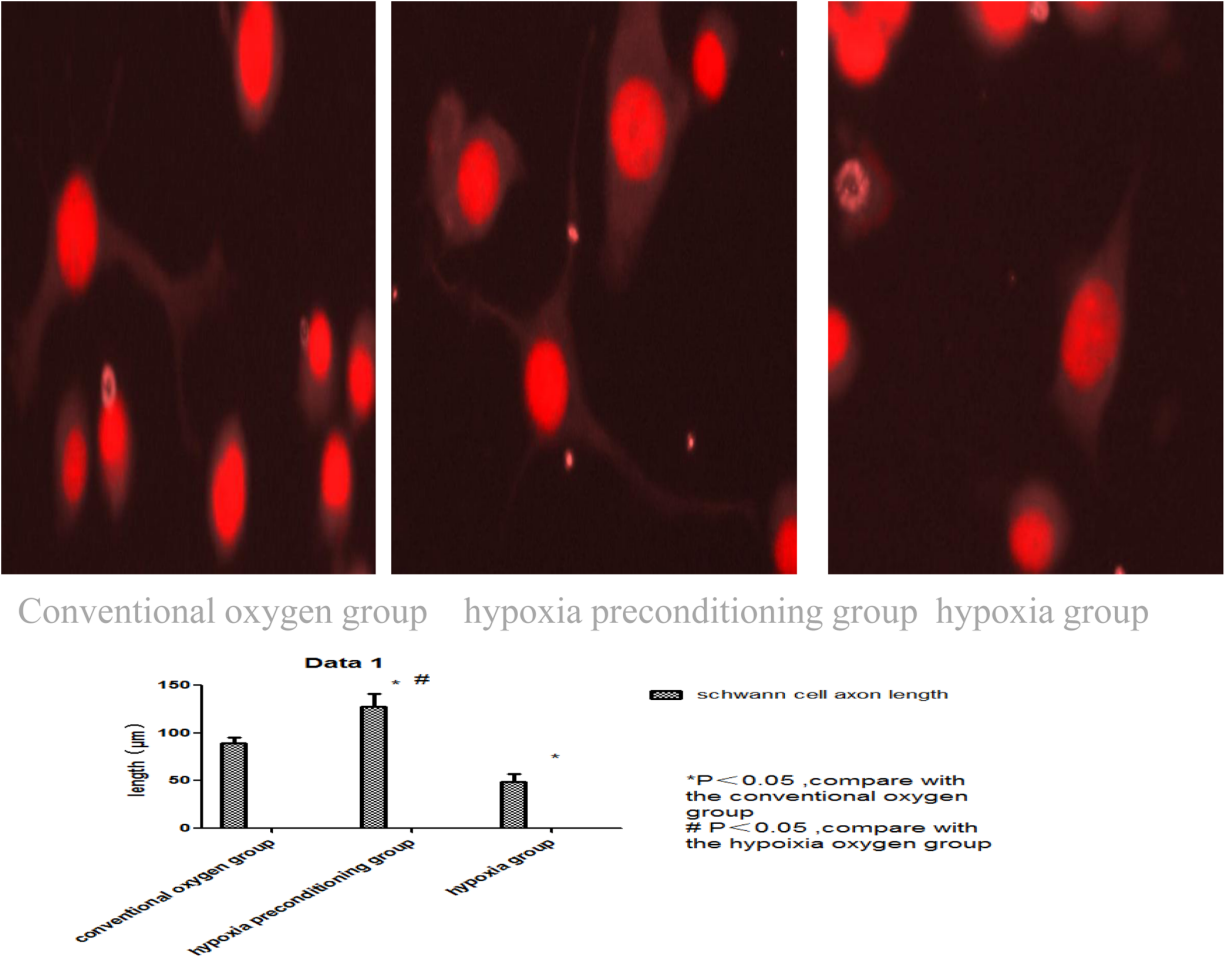
The figure represent the extent of the neurite outgrowth in NG-108 cells in different groups. The figure showed that the NG-108cells in hypoxia-preconditioning group had the longest neurite outgrowth, and the NG-108cells in hypoxia group had the shortest neurite outgrowth. the different among the three group were significant.

When co-cultured with Schwann cells in a hypoxic environment, NG-108 cell axon growth was 128 ± 8.35 μm in the hypoxia-preconditioning group, which was longer than in the conventional oxygen group (90 ±5.28 μm) or the hypoxia group (50± 6.39 μm) (P<0.05), also, NG-108 cell axon growth was longer in the conventional oxygen group (90 ±5.28 μm) than in the hypoxia group (50± 6.39 μm). (P<0.05) (Fig 8, Table 3).

## Discussion

Hypoxic preconditioning is a treatment in which cells are given a brief non-lethal exposure to hypoxia, and it has been shown to result in an improved tolerance of these cells to subsequent exposure to severe hypoxia. Specifically, hypoxia-preconditioned cells can delay apoptosis in response to subsequent hypoxia. The anti-apoptotic capacity of bone marrow stem cells has also been shown to increase in response to hypoxic preconditioning [14,15]. Theus et al. [16] found that hypoxia-preconditioned bone marrow stem cells can promote nerve regeneration in cerebral ischemic rats. Liu and Alkayed [17] demonstrated that astrocytes exhibit better tolerance to serious hypoxia after hypoxic preconditioning. Pacary et al. [18] demonstrated that neurons preconditioned with hypoxia were protected against the effects of subsequent serious oxygen and glucose deprivation. In the current study, electron microscopy analysis revealed that Schwann cells from the conventional oxygen group had an intact nuclear membrane and nucleolus, whereas cells from the hypoxia-preconditioning and hypoxia groups showed varying degrees of damage to these structures. These results indicate that single- or double-stranded DNA breaks occur in Schwann cells under severe hypoxic conditions, whether they were preconditioned or not [19]. The hypoxia-preconditioned Schwann cells also showed partial membrane disintegration, no mitochondrial edema, intact mitochondrial cristae, complete endoplasmic reticulum, and no expansion of the endoplasmic reticulum. These observations demonstrated that these cells were undergoing apoptosis, consistent with the results of the apoptosis analysis, which indicated these cells were in the early stage of apoptosis. By contrast, in the hypoxia group, we found some Schwann cells with ruptured cell membranes, mitochondrial edema, and ruptured mitochondrial cristae, indicating that these cells were necrotic. Taken together, we found that mitochondrial integrity was similar in the conventional oxygen and hypoxia-preconditioning groups, whereas mitochondrial integrity was poor in the hypoxia group. Therefore, mitochondria in cells of the hypoxia group must sustain a certain degree of damage. In this experiment, the hypoxia-preconditioning group exhibited higher mitochondrial membrane potential than the hypoxia group but lower mitochondrial membrane potential than the conventional oxygen group, demonstrating that hypoxic preconditioning can inhibit or delay the decreased mitochondrial membrane potential induced by severe hypoxia but that it cannot completely block these effects. The mitochondrial membrane is a complex structure that plays an important role in the initiation of the apoptotic program. Decreased mitochondrial membrane potential can cause PT pores in the membrane to open [20,21], allowing ions to flow between the inner and outer membranes, disrupting the respiratory chain and inhibiting mitochondrial function. Following these processes, matrix components flow inward, causing the mitochondria to increase in volume and mitochondrial cristae to increase in volume and decrease in surface area, further affect the function of the mitochondria. In addition, these changes to cristae structure can cause the inner membrane to expand more than the outer membrane expansion, causing membrane disintegration, releasing caspase-activating proteins and inducing apoptosis. This was consistent with our experiments, as we found that the mitochondrial membrane potential of Schwann cells was higher in the hypoxia-preconditioning group than in the hypoxia group. SEM results showed that Schwann cells in the hypoxia-preconditioning group had good mitochondrial membrane integrity, mitochondrial cristae swelling without disintegration, whereas Schwann cells in the hypoxia group had significantly decreased mitochondrial membrane potential, incomplete mitochondrial membranes, and disintegration of the mitochondrial cristae. Therefore, although one can still observe defects in mitochondrial structure and function in hypoxic Schwann cells after hypoxic preconditioning, these effects are much less severe than in cells without preconditioning. Using flow cytometry to measure apoptosis, we found that Schwann cells under severe hypoxia conditions following hypoxic preconditioning were primarily in the early stages of apoptosis, whereas Schwann cells under severe hypoxia conditions that had not been preconditioned were primarily in the late stages of apoptosis. By contrast, the conventional oxygen group had many fewer cells undergoing apoptosis compared with the other two groups. These findings demonstrate that hypoxia is a harmful factor that can induce apoptosis and that hypoxic preconditioning can inhibit or delay cell apoptosis. Whether cells in the early stages of apoptosis are committed to cell death is still controversial, although it is generally thought that the process of apoptosis is irreversible. However, there are reports that in the early stage of apoptosis, stimulating factors can reverse this process [22–25]. Therefore, we conclude that under severe hypoxic conditions, Schwann cells that had undergone hypoxic preconditioning had a superior physiological state than Schwann cells that were directly exposed to severe hypoxia.

Bcl-2 family members are classified into one of three functional groups: anti-apoptotic proteins, pro-apoptotic effectors and BH3-only proteins. Bcl-2 proteins are anti-apoptotic proteins, and Bax proteins are pro-apoptotic effectors. Bax oligomers can form channels in the outer membrane, changing mitochondrial permeability and releasing cytochrome C to promote apoptosis. Bax can also bind Bcl-2 protein, reducing the amount of Bcl-2 protein available to inhibit apoptosis. The Bcl-2 protein functions primarily through four pathways [26–28] to inhibit apoptosis: (1) inhibition of glutathione leakage to increase mitochondrial membrane potential; (2) localization of the apoptotic protein precursor Apafl to the mitochondria; (3) neutralization of the Bax protein; and (4) buffering the levels of free calcium ions in the endoplasmic reticulum lumen. Therefore, Bcl-2 and Bax play important antagonistic roles in apoptosis, with Bcl-2 inhibiting apoptosis and Bax promoting apoptosis. The ratio of Bcl-2 to Bax is thus a prime determinant of cell survival or death. When the Bcl-2/Bax ratio is high, the probability of cell survival is also high. By contrast, when the Bcl-2/Bax ratio is low, cells typically exhibit a high probability of apoptosis. However, the effects of hypoxia on apoptosis remain controversial. Riva et al. [29] exposed rats to chronic hypoxia conditions in vivo (10% O_2_), and after three weeks, cell apoptosis was studied in myocardial tissue, fast muscles (gastrocnemius) and slow muscles (soleus) using DNA electrophoresis, TUNEL staining, and quantification of Annexin PV, Bcl-2 and Bax using immunohistochemistry and Western blot methods. Western blot analyses showed that in different tissues under normal and hypoxic conditions, the expression of Bax did not change greatly; Bcl-2 protein levels were higher in fast muscles compared with slow muscles or oxidative muscles; in particular, expression levels were highest in the gastrocnemius muscle, followed by slow muscle, with myocardial tissues showing the lowest expression. After 21 days of exposure to hypoxia, Bcl-2 protein expression was significantly increased in the gastrocnemius (44%), soleus (323%) and heart (1178%) (P<0.05). The researchers were not able to detect apoptosis using the TUNEL method, annexin PV-binding method or DNA electrophoresis analyses. By contrast, Nakamura [30] concluded that short periods of ischemia reperfusion do not change Bcl-2 levels but rather the upregulation of Bax changes the ratio of Bax/Bcl-2 to promote apoptosis. Many studies [31] have shown that hypoxia can induce apoptosis in different cell types, with apoptosis occurring in the oxygen concentration range of 0–0.5%, and not in the range of 1–3%. Differences in the rate of apoptosis are likely due to a variety of factors, including hypoxia degree, duration and testing method. In this experiment, the expression of Bcl-2 in the hypoxia-preconditioning group was higher than that in the group without hypoxic preconditioning, a difference that was significant. In the hypoxia-preconditioning group, the level of Bax expression was higher than that observed in the group that did not receive hypoxic preconditioning, but this effect was not significant. Overall, the ratio of Bcl-2/Bax was higher in the hypoxia-preconditioning group compared to the group that was not exposed to hypoxic preconditioning. Our experiments show that hypoxia can promote expression of the Bax protein, resulting in cell apoptosis. Therefore, in the hypoxia group, Schwann cells directly exposed to severe hypoxia showed increased Bax protein expression without significant changes in Bcl-2 protein levels, leading to a decrease in the Bcl-2/Bax ratio and apoptosis in the Schwann cells. By contrast, following hypoxic preconditioning, increased levels of Bcl-2 lowered the Bcl-2/Bax ratio, inhibiting cell apoptosis. Therefore, in our hypoxia-preconditioning group, both Bcl-2 and Bax protein levels increased but the overall ratio of Bcl-2/Bax decreased, leading to significant anti-apoptotic effects. At the same time, we found that changes in Bax protein expression between the hypoxia and hypoxia-preconditioning groups were not significantly different, suggesting that changes in Bax protein expression are a direct reaction to hypoxic stimulation.

NG-108 cells are fusions of rat glioma and mouse neural cell tumors. There are reports that Schwann cells can promote axonal growth. In this experiment, NG-108 cells and induced Schwann cells were co-cultured under conditions of severe hypoxia. The NG-108 cell axons were longer in the hypoxia-preconditioning group than in the normal oxygen group and the severe hypoxia group. This finding showed that induced Schwann cells are better adapted to function and promote axonal growth in hypoxic environments after hypoxic preconditioning.

## Conclusion

Hypoxic preconditioning can improve the physiological state of Schwann cells in a severe hypoxia environment and improve the ability to promote neurite outgrowth. Overall, our results demonstrate that hypoxic preconditioning is an effective pretreatment method by which to protect induced Schwann cells in hypoxic environments. These results suggest applications for tissue engineering purposes. For example, cells seeded onto grafts often do not establish a mature vascular network when transplanted in vivo, However, hypoxic preconditioning could improve the ability of such cells to withstand negative hypoxic conditions in the transplant environment. Such hypotheses will require further investigation.

## Supporting Information

S1 Table The data for mitochondrial membrane potential result. (DOCX)

S2 Table The data for Cell apoptosis flow cytometry results. (DOCX)

S3 Table The data for Western-Blot assay of Bax,Bcl-2 and Bcl-2/Bax. (DOCX)

S4 Table The data for the length of Schwann cell axon growth. (DOCX)
