# Supplementary material for: The Effect of Hypoxic Preconditioning on Induced Schwann Cells under Hypoxic Conditions
Source: PLoS One. 2015 Oct 28;10(10):e0141201. doi: 10.1371/journal.pone.0141201 (PMC4624905; doi:10.1371/journal.pone.0141201)
Supplement: S1 Table — (DOCX) [file pone.0141201.s001.docx]

1. S1 Table the data for mitochondrial membrane potential result

| Group | Data1 | Data2 | Data3 |
| --- | --- | --- | --- |
| Conventional oxygen | 94.10 | 94.00 | 94.40 |
| Hypoxia preconditioning | 89.90 | 89.70 | 90.50 |
| Hypoxia | 80.90 | 80.10 | 79.90 |
