# Supplementary material for: The Effect of Hypoxic Preconditioning on Induced Schwann Cells under Hypoxic Conditions
Source: PLoS One. 2015 Oct 28;10(10):e0141201. doi: 10.1371/journal.pone.0141201 (PMC4624905; doi:10.1371/journal.pone.0141201)
Supplement: S2 Table — (DOCX) [file pone.0141201.s002.docx]

S2 Table the data for Cell apoptosis flow cytometry results

| Group | Early stage of apoptosis | | | Later stage of apoptosis | | | Necrosis | | |
| --- | --- | --- | --- | --- | --- | --- | --- | --- | --- |
| Conventional oxygen | 0.403 | 0.155 | 0.360 | 0.272 | 0.175 | 0.138 | 0.867 | 0.63 | 0.795 |
| Hypoxia preconditioning | 82.400 | 80.800 | 80.200 | 16.600 | 16.100 | 19.200 | 0.441 | 0.254 | 0.138 |
| Hypoxia | 0.185 | 0.270 | 0.250 | 31.700 | 35.200 | 35.100 | 61.400 | 60.700 | 59.20 |
