# Supplementary material for: The Effect of Hypoxic Preconditioning on Induced Schwann Cells under Hypoxic Conditions
Source: PLoS One. 2015 Oct 28;10(10):e0141201. doi: 10.1371/journal.pone.0141201 (PMC4624905; doi:10.1371/journal.pone.0141201)
Supplement: S3 Table — (DOCX) [file pone.0141201.s003.docx]

S3 Table the data for Western-Blot assay of Bax,Bcl-2 and Bcl-2/Bax

| Group | Bcl-2 | | | Bax | | | Bcl-2/Bax | | |
| --- | --- | --- | --- | --- | --- | --- | --- | --- | --- |
| Conventional oxygen | 0.62 | 0.75 | 0.68 | 0.75 | 0.89 | 0.70 | 0.82 | 0.84 | 0.98 |
| Hypoxia preconditioning | 2.28 | 2.05 | 2.08 | 0.95 | 1.03 | 1.07 | 2.68 | 1.78 | 1.53 |
| Hypoxia | 0.45 | 0.49 | 0.38 | 1.02 | 0.99 | 0.85 | 0.41 | 0.50 | 0.44 |
