# Supplementary material for: The Effect of Hypoxic Preconditioning on Induced Schwann Cells under Hypoxic Conditions
Source: PLoS One. 2015 Oct 28;10(10):e0141201. doi: 10.1371/journal.pone.0141201 (PMC4624905; doi:10.1371/journal.pone.0141201)
Supplement: S4 Table — (DOCX) [file pone.0141201.s004.docx]

S4 Table the data for the length of Schwann cell axon growth

| Group | Schwann cell axon length | | |
| --- | --- | --- | --- |
| Conventional oxygen | 96 | 84 | 89 |
| Hypoxia preconditioning | 126 | 142 | 114 |
| Hypoxia | 57 | 41 | 49 |
